# Chickpea and Lupin Sprouts, Stimulated by Different LED Lights, As Novel Examples of Isoflavones-Rich Functional Food, and Their Impact on Breast and Prostate Cells

**DOI:** 10.3390/molecules27249030

**Published:** 2022-12-18

**Authors:** Agnieszka Galanty, Paweł Zagrodzki, Marina Miret, Paweł Paśko

**Affiliations:** 1 Department of Pharmacognosy, Faculty of Pharmacy, Medical College Jagiellonian University, Medyczna 9, 30-688 Kraków, Poland; 2 Department of Food Chemistry and Nutrition, Faculty of Pharmacy, Medical College Jagiellonian University, Medyczna 9, 30-688 Kraków, Poland; 3 Faculty of Pharmacy and Food Science, University of Barcelona, Campus Diagonal, Av. de Joan XXIII 27-31, 08028 Barcelona, Spain

**Keywords:** isoflavones, LED light, sprouts, breast cancer, prostate cancer, legumes

## Abstract

Among all legumes sprouts’ active compounds, isoflavones seem to be the most important; nevertheless, their high content is not always associated with beneficial effects. These compounds may prevent or stimulate hormone-dependent cancers due to their estrogen-like activity. Different LED light quality can change the synthesis of active compounds and significantly influence the biological activity of the sprouts. This study aimed to evaluate the effects of LED light (red, blue, green, yellow), as well as total darkness, and natural light conditions (as reference), on isoflavones content, determined by HPLC-UV-VIS, during 10 days of harvesting of chickpea and lupin sprouts. Due to the ambiguous estrogenic potential of isoflavones, the impact of these sprouts on normal and cancer prostate and breast cells was evaluated. Yellow LED light resulted in the highest sum of isoflavones in chickpea sprouts (up to 1 g/100 g dw), while for green LED light, the isoflavones sum was the lowest. The exact opposite effect was noted for lupin sprouts, with the predominance of green over the yellow LED light. The examined sprouts were of high safety to non-neoplastic breast and prostate cells, with interesting cytotoxic effects on breast MCF7 and prostate DU145 cancer cells. No clear relationship was observed between the activity and isoflavones content.

## 1. Introduction

There is a large variety of generally available sprouts, among which legumes (e.g., soybean, mung bean, alfalfa, clover, etc.) sprouts are most commonly consumed. These sprouts are a rich and valuable source of different nutrients, such as proteins, dietary fiber, essential and trace elements, and vitamins (especially B group) [1], which may have a significant impact on human health, especially in the case of vegans and vegetarians. As a food product, sprouts differ significantly in terms of concentrations and the quality of bioactive compounds from seeds and other parts of plants, recognized as conventional vegetables (leaves, roots, bulbs, florets). It is known that sprouts, as the examples of new vegetables, are also a good source of various bioactive compounds, which can be responsible for their beneficial effect in the prevention of different diseases, including cancer. Until now a lot of studies in different in vitro models have confirmed this effect, resulting mostly from the abundance of polyphenolic compounds [2], including our previous studies on broccoli or clover sprouts [3,4]. The nutritional and sensory properties of legume seeds can be improved by the germination process [1]. In the case of legumes sprouts and their active compounds (phenolic compounds, phytates, saponins, isoflavones, lectins), isoflavones seem to be the most important; nevertheless, their high content is not always associated with beneficial effects. Isoflavones were also recognized as a potential element in the stimulation of hormone-dependent cancers, due to their estrogen-like activity [3]. Several in vitro studies on legumes sprouts have shown various beneficial properties, including antioxidant, anti-diabetic, antimicrobial, anti-hyperlipidemic, antihypertensive, anti-inflammatory, anticancer, and anti-mutagenic [5,6]. Farag et al. [7] suggested that legumes sprout extracts are potential sources of nutrients and may be used as dietary supplements or functional foods. Observational studies in humans related to legume consumption are limited. Some studies report that beans may be inversely associated with advanced adenoma recurrence; legume intake also decreases the risk of developing colon cancer [2].

Manipulation of light quality is one of the interesting strategies used to increase or decrease the synthesis of beneficial or harmful compounds in sprouts. It is known that wavelength, intensity, duration, and the direction of light are significant factors for sprouts to grow [8]. Effective harvesting modification, associated with total darkness, was successfully proven in our previous study with brassica sprouts [9]. Due to the variety of effects of using different light sources, like LED, halogen, fluorescent or high-pressure sodium lamps, influencing the nutrient level in the sprouts, such an experimental approach seems to be important for the future development of vegetable production. This may result in obtaining the vegetables with a defined and controlled level of bioactive compounds, as an answer to the personalized demand for functional foods, indicated by people with special dietary requirements and/or increased risk of different diseases, including hormone-dependent cancers [10].

Recently, an increased number of studies have demonstrated the beneficial effects of LED light on plant growth and the quality of crops, including the accumulation of phytonutrients in sprouts and microgreens [11,12]. Notably, compared with conventional light sources, LED light is regarded as a cheap, cool, and controllable one that can selectively and quantitatively provide different spectra. This light may activate different biochemical pathways to influence plant growth in such aspects as leaf area, thickness, stem length, and active compounds quality and quantity via photoreceptors (phytochrome and cryptochrome) [13,14]. Blue- and red-light regions are most efficiently absorbed by chlorophylls during the photosynthetic processes [15]. Consequently, these two light spectra have been mostly studied in plant photobiology. Several previously published investigations reported that LED light can improve the anti-oxidant properties of sprouted seeds (e.g., lentils, wheat, and radish) [16]. However, the effects of different types of LED lamps on the levels of phenolic compounds in legume sprouts have been poorly studied, and they were described only in a few species, like alfalfa, clover, or soy [3,17,18]. Therefore, the objectives of our study were to evaluate the effects of various types of LEDs, including red, blue, green, and yellow light, as well as total darkness, in relation to natural light conditions as the reference, on the composition and amount of isoflavones during the monitored 10-day period of harvesting of the selected legume sprouts. Six species of edible legume seeds were chosen for the study: lupin (*Lu-pinus luteus* L.); chickpea (*Cicer arietinum* L.); common vetch (*Vicia sativa* L.); lentil (*Lens culinaris* Medik.); bird’s-foot (*Ornithopus sativus* Brot.); and lotus (*Lotus corniculatus* L.). Four of them are not typical for sprouts production (common vetch, bird’s-foot, lupin, lotus) and generally used in agriculture to produce fodders and silages, but until now have not been implemented as functional foods. Two of them (chickpea, lentil) are well known mostly because of their value in human nutrition as a seed source in the diet, with a limited position as sprouts on the food market and scarce evaluation on their sprout functional potential and usefulness in a daily menu. Due to the ambiguous estrogenic potential of isoflavones in legume vegetables, it was also decided to evaluate the possible impact of the sprouts on non-neoplastic breast and prostate cells, but also on hormone-dependent breast and prostate cancer cells at different stages of malignancy and different expressions of estrogen and androgen receptors. This is a novel idea in the evaluation of the possible potential of functional foods and their preselection in terms of safety and efficacy.

## 2. Results and Discussion

### 2.1. Qualitative Isoflavones Analysis

The first step of the project was associated with the preselection of the best, mostly new, legumes sprouts, which can be a novel source of isoflavones in the daily diet. Qualitative analysis of the evaluated seeds and sprouts (lupin, chickpea, common vetch, lentil, bird’s-foot, lotus) allowed to identify isoflavones only in two of the examined sprouts species, namely chickpea and lupin. Moreover, isoflavones were also observed in the seeds of the latter species, while in chickpea seeds only traces of isoflavones were noted. None of the tested sprouts and seeds revealed the presence of daidzein. Some qualitative differences in the isoflavones composition of chickpea and lupin sprouts were observed. In chickpea sprouts, the predominance of ononin, biochanin A, and formononetin was noted, but daidzein, genistein, and glycitein were also present. The three predominant isoflavones were also observed in chickpea sprouts by other authors [18,19,20], while the presence of daidzein, genistein, and glycitein was described here for the first time. In the case of lupin sprouts, genistein and glycitein were predominant isoflavones, followed by genistein and biochanin A. Genistein and its derivatives were also described by Duenas et al. [21] in 9-day lupin sprouts, while the presence of the other mentioned isoflavones was described in our study for the first time. Based on the results of the qualitative analysis, only the seeds and sprouts of chickpea and lupin were further evaluated in aspects of the quantitative isoflavones analysis and cytotoxic potential.

### 2.2. Chickpea and Lupin Seeds—Isoflavones Content and Cytotoxicity

**Figure 1:**
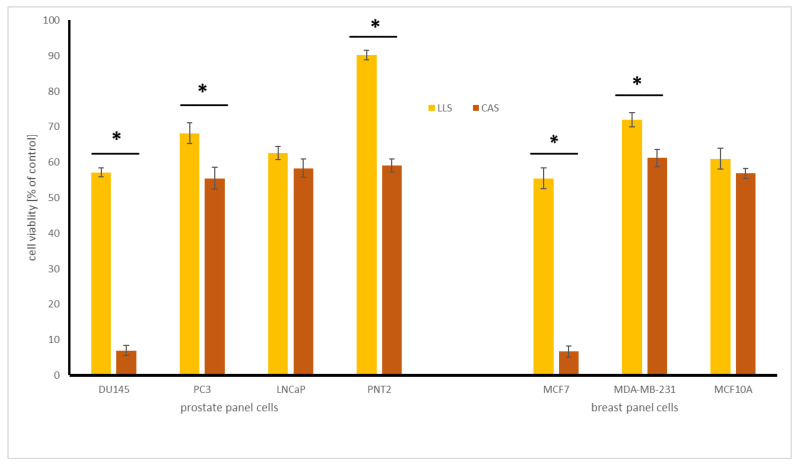
Cytotoxic effect of lupin (LLS) and chickpea (CAS) seed extracts on prostate and breast normal and cancer cells (for the acronyms of the cell lines–see Section 2.5). Cells were treated with 500 µg/mL of seed extracts (n = 3) for 24 h. Values are presented as the mean ± SD (standard deviation). Significant differences between two kinds of seeds (*p* < 0.05) were marked with *.

Quantitative HPLC analysis showed isoflavones (mg/100 g dw) in lupin seeds (LLS) with the highest level of genistein (14.2 ± 2.0), followed by genistein (0.34 ± 0.02), glycitein (0.14 ± 0.05), and biochanin A (0.08 ± 0.02). In the case of chickpea seeds (CAS), the above-mentioned isoflavones were found only in traces. Mazur et al. [22] showed that the highest total concentration of isoflavones in edible legume seeds was in soybeans (ranging from 37.3 to 140.3 mg/100 g) and chickpea (1.1 to 3.6 mg/100 g). The pea “Green split” was found to be the poorest source of these compounds. Kahn et al. [23] noted that in different lupin seeds and their byproducts, genistein and its derivatives were the major isoflavones, which is in agreement with our observation, in which genistein (glycoside of genistein) was the dominant compound. The cytotoxic effect of extracts of chickpea and lupin seeds on prostate and breast normal and cancer cells was presented in Figure 1.

The results indicate that CAS extract was significantly more active in comparison with LLS extract towards all the tested cell lines, with the exception of androgen-dependent LNCaP and non-neoplastic MCF10A cells. The CAS extract affected the viability of DU145 prostate cancer and MCF7 breast cancer cells, with IC_50_ 145.0 and 154.1 µg/mL, respectively, while it was toxic to non-neoplastic PNT2 and MCF10A cells to a much lesser extent. In a similar study, no cytotoxic activity of lupin seeds extract was noted against MCF-7 and PC3 cells, up to 1 mg/mL [23], while our results indicate a decrease in cell viability treated with LLS extract to about 65 and 55%, respectively, at the dose of 0.5 mg/mL. No data can be found on the cytotoxic impact of chickpea seeds.

### 2.3. Influence of Light Quality on the Isoflavones Content in Sprouts

**Figure 2:**
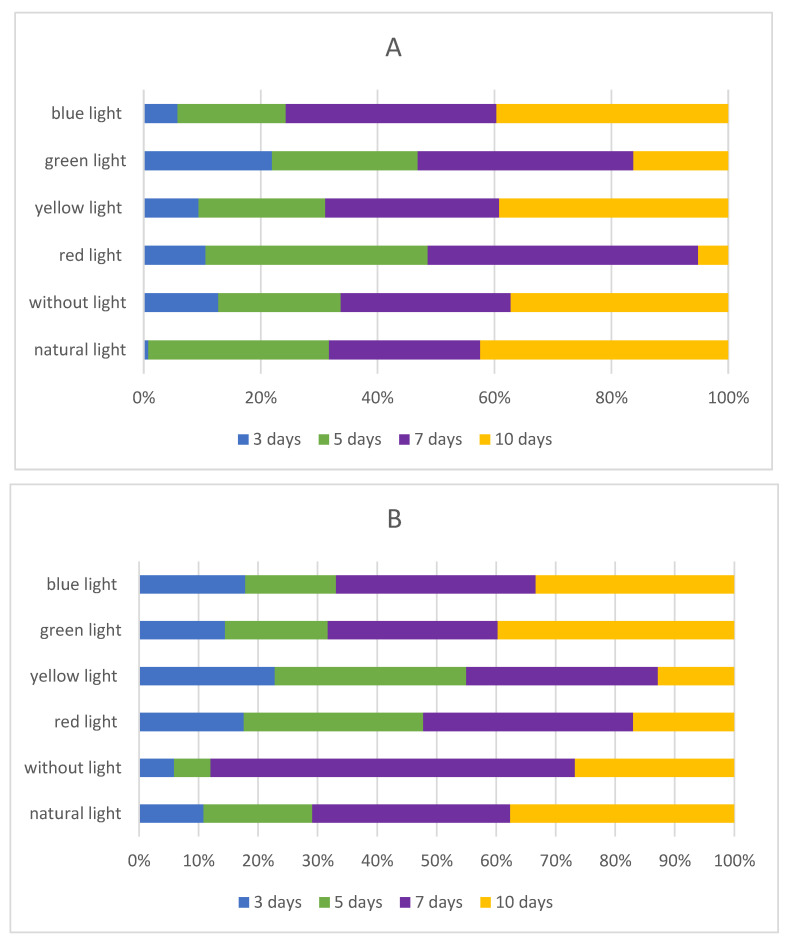
Cumulation dynamics of isoflavones sums [%] in chickpea (**A**), and lupin (**B**) sprouts harvested for 3, 5, 7, and 10 days.

Isoflavones are formed during germination through the malonate and phenylpropanoid pathways [24], and the increase in their content, compared with flavones and phenolic acids, is rapid. Because of this, in the next step of our investigation, we decided to evaluate if there is any influence of LED light quality on the synthesis and content of isoflavones during the sprouting process. The detailed amount of isoflavones in chickpea sprouts and lupin sprouts is shown in Table 1 and Table 2, respectively. Additionally, total isoflavones in sprouts are shown in Figure 2.

Chickpea and lupin sprouts harvested in natural light conditions were used in this study as reference materials. In CAL sprouts, three isoflavones were found in high concentration (biochanin A, formononetin, ononin), while for LLL sprouts, genistein and glycitein were dominant compounds. Moreover, in CAL sprouts, the contents of biochanin A, daidzein and formononetin were increasing during sprouting time, while the other three isoflavones were synthesized in different modes (Table 1). In LLL, the contents of biochanin A and genistein were increasing throughout the harvesting time, with glycitein being synthesized in a rise-and-fall mode, and no genistein present (Table 2). For better visualization of the changes in the isoflavones level during the sprouting time and different impact of the light treatments, extra diagrams were attached in the Supplementary Material; Figures S1 and S2 for chickpea and lupin sprouts, respectively.

#### 2.3.1. Influence of Darkness on the Isoflavones Content in Sprouts

The total lack of light brought about varied, significant effects on the content of individual isoflavones in CAN and LLN sprouts extracts, and this effect was also dependent on the day of sprouting, with visible fluctuation, especially in LLN, in comparison to LLL. In CAN sprouts, biochanin A, daidzein, formononetin, and glycitein contents were constantly increasing but further decreased in CA10N, significantly for formononetin. A similar stable increase was noted for ononin up to the 7th day, but with a rapid, almost fourfold increase in CA10N. In the case of LLN sprouts, darkness caused an increase in biochanin A and glycitein synthesis during sprouting up to the 7th day, followed by a decrease in the last day of sprouting, especially significant in the case of biochanin A. The same effect was also observed for genistein (LL7N vs. LL10N). It should be noted that genistein started to be synthesized from the 7th day and increased significantly up to the 10th day, while the compound was not observed in LLL sprouts.

The sum of isoflavones in CA10N sprouts, when compared with CA10L sprouts, was significantly lower (559.6 vs. 791.3 mg/100 g dw, respectively), because of the general inhibition of isoflavones synthesis after the 7th day of sprouting. For lupin sprouts, it should be noted that in LL3N and LL10N sprouts, there were no significant changes in the total sum of isoflavones when compared to respective LLL sprouts.

Light exposure was previously reported to boost isoflavones content in soybean and chickpea sprouts [17,18]. An increase in isoflavones content was observed when soybeans were germinated in the presence of light. This phenomenon was explained as the influence of light rays on the production of malonyl-CoA and coumaroyl-CoA, thus enhancing the pool size of natural precursors of isoflavones, including daidzein [25], which was also confirmed in our study. Moreover, an increase in the content of these compounds can be stimulated also by the pathways of naringenin chalcone and isoliquiritigenin, the precursors of isoflavones in legumes [26]. Gao et al. [18] noted that during germination, the contents of formononetin and biochanin A increased; the maximum amount was obtained on day 10, which is a similar observation to our study. Our results suggest that generally formononetin, followed by biochanin A, are the predominant isoflavones in chickpea sprouts, but in 10-day sprouts ononin started to be the dominant compound and it is consistent partially with the results of Gao et al. [18] and Ma et al. [27]. Gao et al. [18] observed a significant decrease of genistein in chickpeas during sprouting time, similar to our study. This can be possibly explained by the decrease in the hydrolysis of glycosides through β-glucosidase during germination. Zhang et al. [8] found that alfalfa sprouts, germinated in the light-exposure, have higher content of isoflavones, and it was associated with different expressions of genes involved in the daidzein and genistein biosynthesis pathway (HIDH, HI4OMT, IF7GT, IF7MAT, CYP81E, CYP93A1).

Cultivation of legume sprouts, i.e., soybean, under darkness conditions has been used in agriculture to improve sprout quality, e.g., the increase in hypocotyl length, decrease in the formation of unflavored taste, and relatively soft texture [28]. However, there are only a few reports on the influence of the darkness on the isoflavones level in the sprouts. Kirakosyan et al. [29] found that the isoflavones level was enhanced in dark-grown soybean, compared with the light-grown plants in three of the five genotypes of soy, which indicates that phytochrome reactions are strongly dependent on the genotypes of plants. Graham [30] noticed that growth in continuous darkness significantly affects not only the level but also the distribution of the isoflavones in soybean tissues. The levels of daidzein, genistein, and their conjugates were significantly higher in dark- versus light-grown cotyledons and their levels reduced in all other dark-grown seedling tissues. During harvesting of the sprouts in the darkness, we noted some unexpected results in the increasing sum of isoflavones and selected compounds. This may result from the use of other species in our study, as the above-mentioned results were performed only on soy. Moreover, as the data on the influence of harvesting sprouts in the darkness is scarce, further studies are needed to explain the mechanisms of the obtained effects. These may involve the activity of different enzymes such as: phenylalanine ammonia lyase (PAL), cinnamic acid 4-hydroxylase (C4H), enzyme 4-coumarate: coenzyme A ligase (4CL), and more specifically like: chalcone synthase (CHS) chalcone reductase (CHR), chalcone isomerase (CHI), isoflavone synthase (IFS), isoflavone dehydratase (IFD). Additionally, isoflavone 4′-O-methyltransferase (IOMT) activity should be also evaluated because genistein and daidzein get converted to biochanin A and formononetin [31].

#### 2.3.2. Influence of Red LED Light on Isoflavones Content in Chickpea and Lupin Sprouts

Red LED light had significant effects on the content of isoflavones. Generally, in chickpea sprouts, the amounts of all isoflavones increased during sprouting time up to the 7th day breakthrough point, manifested as a significant decrease, or even lack of, in the case of genistein. For lupin sprouts, red LED light had a diverse effect on the isoflavones synthesis during sprouting, with no similarities to LLL. The trends in biochanin A and genistein synthesis were similar, with a breakthrough point and decrease on the 7th day, as was also observed in chickpea sprouts. The opposite pattern was noted for genistein and glycation, with the lowest amounts of the compounds in LL5R sprouts, followed by a further increase.

The dramatic reduction in dominant compounds (biochanin A, formononetin, and ononin) content caused a significant drop in the sum of isoflavones in CA10R versus reference CA10L sprouts (86.6 vs. 791.3 mg/100 g dw), which indicates that during harvesting of sprouts in red LED light, the time of the exposition is crucial for the synthesis of these compounds. It was also confirmed for lupin sprouts, where the total amount of isoflavones in LL10R sprouts significantly decreased in comparison with LL7R. Additionally, this parameter did not differ significantly for LL10L or LL10R (87.4 vs. 72.6 mg/100 g dw).

It is the first study that clearly indicates the effect of red LED light on isoflavones synthesis in any legume sprouts. In the case of *Brassica juncea* sprouts, red LED light altered the production of 4-hydroxyglucobrassicin, 4-methoxyglucobrassicin, glucoiberin, gluconapin, glucobrassicin, gluconasturtiin, sinigrin, and neoglucobrassicin when compared with white and blue LED lights [32]. It was also noted that red LED light increased hypocotyl and sprout length, decreased microbial growth, and improved the antioxidant activities, compared with darkness and fluorescent lighting treatments, but did not stimulate the biosynthesis of phenolic acids in broccoli sprouts [33].

There is some positive evidence that red LED light can promote photosynthesis and plant growth (content of chlorophyll, formation of photosynthetic apparatus, inducing stomatal opening) [34,35]. Zhen et al. [36] suggested that far-red light, usually considered as photosynthetically inefficient radiation, can be more effective in improving photochemical productivity than it was previously believed. This is associated with the synergistic effect between far-red light and light with shorter wavelengths [37]. Under red light, stem elongation is slow enough so that the changes in auxin availability or sensitivity could mediate the response [38]. But also, negative feedback should be highlighted that prolonged red-light exposure may cause the “red light syndrome”, characterized by lowering photosynthetic capacity, maximum quantum yield of chlorophyll fluorescence, carbohydrate accumulation, and reduced growth [39]. It was also reported that the stem growth of pea seedlings was inhibited by red light via decreasing the cell-wall yield coefficient [38]. Further study of the influence of red light seems to be an interesting new direction, in terms of plant production and sprouts harvesting.

#### 2.3.3. Influence of Yellow LED Light on Isoflavones Content in Chickpea and Lupin Sprouts

Yellow LED light revealed the best effect on the synthesis of isoflavones in chickpea sprouts. The content of all examined compounds increased during sprouting in yellow LED light, except for ononin, which then slightly decreased for 7th day sprouts, but the difference was insignificant. The steady increase pattern of isoflavones synthesis is highly important in the case of glycitein, genistein, and especially ononin, as their synthesis in the reference CAL sprouts harvested in the natural light conditions was rather in a decrease-increase mode. The opposite effect was observed for LLY sprouts, in which the synthesis of isoflavones was changed from the increasing pattern (in reference LLL sprouts) to increase-decrease mode for biochanin A and genistein. Notably, for glycitein, the trend shifted to more stable growth, when compared with the reference LLL sprouts. This may suggest that the effect of yellow LED light strongly depends on the genus of seeds used for sprouting.

Yellow LED light caused a significant increase in the sum of isoflavones in CA10Y, in comparison with CA10L sprouts (953.9 vs. 791.3 mg/100 g dw). For lupin sprouts, yellow LED light had a negative effect on the isoflavones sum during sprouting (LL10L 87.4 vs. LL10Y 42.6 mg/100 g dw). The lowest amount of isoflavones was noted in LL3Y and LL10Y sprouts.

No studies have been performed so far describing the influence of yellow LED light on isoflavones synthesis. In similar studies, yellow LED light increased total carotenoids content of tatsoi microgreens and chickpea sprouts [40,41]. This type of light in brassica microgreens (mizunas, pak choi, red radish, white mustard) improved the glucosinolates content [42], which can be associated with the activation of different enzymes involved in their synthesis (MAM1, CYP79F1, CYP83A1, CYP83B1, SUR1, UGT74B1, MAM1, CYP79F1, CYP83A1) [43].

#### 2.3.4. Influence of Green LED Light on Isoflavones Content in Chickpea and Lupin Sprouts

Green LED light had the most variable influence effect on the synthesis of isoflavones in chickpea sprouts, with significant fluctuations. In comparison with other light types evaluated in our experiment, green LED light modified the quantitative relations of particular isoflavones, with ononin no longer the predominant isoflavone in CAG sprouts, and biochanin A as a leading compound. The traces of genistein in CA3G, CA5G and daidzein in CA3G in comparison with CAL should also be noted. For lupin sprouts, green LED light had a completely reversed effect, with a significant increase in the amounts of all isoflavones during sprouting, except for genistein. This range of light significantly improved the content of biochanin A and especially glycitein in LLG sprouts, in comparison with the reference LLL material. What should be highlighted is that green LED light caused a significant increase in the synthesis of genistein, which became one of the predominant isoflavones in LL10G, while the compound was only found in LLL in traces.

The sum of isoflavones in green LED light in CA10G sprouts significantly dropped vs. reference CA10L sprouts (114.5 vs. 791.3 mg/100 g dw), with significant differences observed also between CA3G (154.2 mg/100 g dw) and CA10G (114.5 mg/100 g dw) sprouts. For lupin sprouts, green LED light caused a reverse effect, with a significant increase in the total sum of isoflavones in LL10G, in comparison to the reference LL10L sprouts (87.4 vs. 125.9 mg/100 g dw).

The results obtained for chickpea sprouts were as expected. It is known that green light is weakly absorbed by chlorophylls [10]. The results for lupin sprouts are noteworthy for further study, and in agreement with Smith et al. [44], who indicated that this kind of light should not be neglected in agriculture. Green light can penetrate the leaf further than blue and red lights, which improves assimilation of CO_2_ and promotes higher biomass and yield. The mentioned processes are a crucial signal for long-term developmental and short-term dynamic acclimation to the environment [44]. Kim et al. [45] suggested that green light improved plants appearance, which helps to visualize pests, disease, or nutrient deficiency in plants. Kwack et al. [38] also found the positive impact of green light on the nutritional status of legume sprouts, such as alfalfa and clover.

#### 2.3.5. Influence of Blue LED Light on Isoflavones Content in Chickpea and Lupin Sprouts

Blue LED light had varied effects on the content of individual isoflavones. In chickpea sprouts, the content of daidzein and ononin increased up to the 7th day of sprouting, followed by a decrease, and the differences were significant. On the contrary, an increase in the level of biochanin A and glycitein in CA10B vs. CA7B sprouts was noted. Blue LED light also caused inhibition of genistein synthesis in all CAB sprouts. Notably, the synthesis pattern, observed as a steady increase, was similar for biochanin A, formononetin and glycitein, while daidzein and ononin synthesis was characterized by a decrease in CA7B sprouts. Moreover, for biochanin A and glycitein synthesis in lupin sprouts, the pattern was similar to that observed in chickpea sprouts. Notably, blue LED light stimulated the synthesis of genistein in LLB sprouts in an increasing mode, while the compound was observed in the reference LLL sprouts only in traces.

The total sum of isoflavones in CA10B sprouts dropped significantly, in comparison with CA10L sprouts (563.4 vs. 791.3 mg/100 g dw); the same tendency was observed for lupin sprouts.

Azad et al. [46] noted that isoflavones (daidzein, glycitein, genistein, daidzein, genistein) were more efficiently accumulated in the five- and six-days soybean sprouts grown under blue LED light, compared with the green and fluorescent light. A reduction in the isoflavones contents was observed with increasing sprouting time, which is mostly an opposite effect to that in our study on the influence of blue light on legume sprouts. Cevallos-Casals et al. [47] indicated that blue light is the most effective lighting source for the synthesis of flavonoid compounds by stimulating phenylalanine ammonia-lyase, chalcone synthesis, and dihydroflavonol-4-reductase gene expression. Blue light was reported to increase the chlorophyll content, promote stomatal opening, and control the integrity of chloroplast protein [48].

### 2.4. Cytotoxic Activity of Chickpea and Lupin Sprouts Harvested in Different Light Quality

In the next step of the experiment, we examined the cytotoxic potential and safety of the tested sprouts, with reference to their varied isoflavones content and estrogenic properties. Thus, two cellular models, namely breast and prostate panels, were prepared for this purpose, comprising non-neoplastic and cancer cells, differing in their response to estrogens, but also metastatic properties. The breast panel consisted of low invasive, estrogen- and progesterone-receptor positive MCF7, and highly metastatic, estrogen- and progesterone-receptor negative MDA-MB-231 breast cancer cells, completed with non-neoplastic breast epithelial MCF10A cells. The prostate panel included androgen-dependent LNCaP, and androgen-insensitive DU145 and PC3 prostate cancer cells, with low and high metastatic potential, respectively, completed with non-neoplastic prostate epithelial PNT2 cells.

#### 2.4.1. Influence of Chickpea and Lupin Sprouts on Breast Cells’ Viability

**Figure 3:**
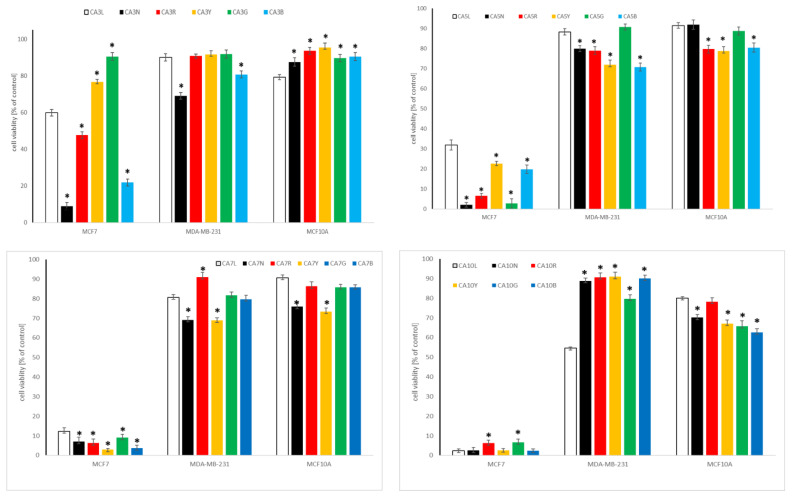
Cytotoxic effect of the extracts of chickpea sprouts (CA) harvested for 3, 5, 7, and 10 days in different LED light conditions: natural (L), darkness (N), red (R), yellow (Y), green (G), blue (B) on breast normal (MCF10A) and cancer (MCF7, MDA-MB-231) cells. Cells were treated with 500 µg/mL of sprout extracts (n = 3) for 24 h. Values are presented as the mean ± SD (standard deviation). Significant differences (*p* < 0.05) for each cell lines refer to normal sprouts harvesting procedure and are marked as *.

**Figure 4:**
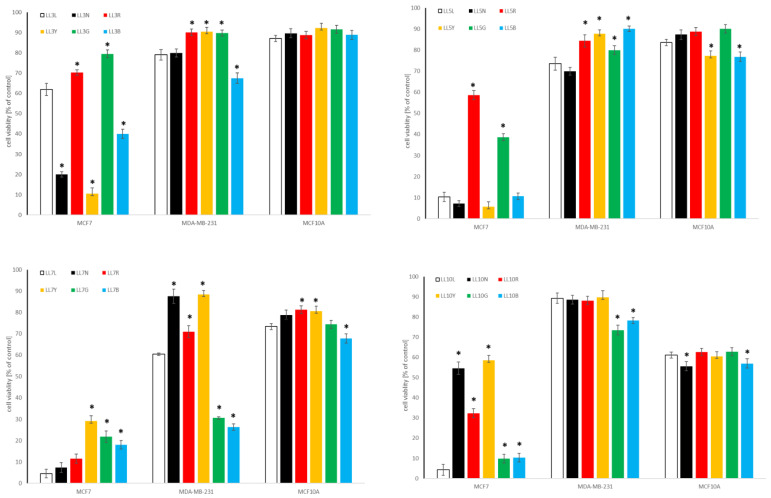
Cytotoxic effect of the extracts of lupin sprouts (LL) harvested for 3, 5, 7, and 10 days in different LED light conditions: natural (L), darkness (N), red (R), yellow (Y), green (G), blue (B) on breast normal (MCF10A) and cancer (MCF7, MDA-MB-231) cells. Cells were treated with 500 µg/mL of sprout extracts (n = 3) for 24 h. Values are presented as the mean ± SD (standard deviation). Significant differences (*p* < 0.05) for each cell line refer to normal sprouts harvesting procedure and are marked as *.

Both chickpea and lupin sprouts revealed a varied impact on the tested cells of breast panel, and the results are presented in Table 3 as IC_50_, and on Figure 3 and Figure 4, for the highest tested concentration of 500 µg/mL. Estrogen- and progesterone-receptor positive MCF7 cells were the most susceptible among the cell lines tested, with IC_50_ values as low as 35 (CA10L), 42.1 (CA5N) or 46.8 (CA10B) µg/mL for the three most active extracts. Although according to the criteria of the National Cancer Institute and Geran protocol [49] for the extracts, this activity is classified as moderate cytotoxicity; it should be underlined that in the case of sprouts extracts such high activity is rarely observed, with most of the published IC_50_ exceeding 300 or even 500 µg/mL [50,51]. Notably, although the most active CA10L, CA5N, and CA10B sprouts extracts were rich in isoflavones (sum > 500 mg/100 g dw), the extract with the highest isoflavones sum (CA10Y) was less active. Thus, we cannot draw the final conclusions on the relationship between isoflavones content in CA sprouts and their cytotoxicity to MCF7 cells, although some tendency appeared, as the sprouts with a low isoflavones amount were in most cases also less active. In the case of lupin sprouts, their activity to MCF7 cells was much lower, with IC_50_ in a range from 77.2–424.1 µg/mL. Similarly to chickpea, 10-day lupin sprouts harvested in normal light conditions were most active to MCF7 cells.

Metastatic and hormone-independent MDA-MB-231 cells were highly resistant to the influence of the tested extracts, with IC_50_ exceeding the highest tested concentration. The two exceptions were LL7G and LL7B sprouts, with IC_50_ 320.6 and 307.5 µg/mL, respectively. To verify the safety of the tested sprouts, we determined their activity also to non-neoplastic breast epithelial cells MCF10A. At the highest tested concentration of 500 µg/mL, the CA and LL sprouts extracts were only slightly toxic, with a cell viability decrease up to 60% for 10-day sprouts. This indicates good selectivity and high safety of the tested samples.

Only a few studies so far indicated the cytotoxic potential of legumes sprouts to breast cancer cells. Two of them concerned the activity of isoflavones fraction from chickpea sprouts to MDA-MB-231 and MCF7 cells, respectively, and their results indicated a cytotoxic effect at doses above 1, or even 11 mg/mL [19,20], which is much weaker activity than in our study. A study on cytotoxic activity of fenugreek sprouts was also performed, with IC_50_ > 500 µg/mL [50]. In our recently published study, cytotoxic activity of the sprouts of four clover species was described on the same breast panel, with IC_50_ ranging from 61.1–361.3 (MFC7 cells), 56.7–457.5 (MDA-MB-231 cells), and 315.7–440.6 (MCF10 cells) µg/mL, which is similar to the observations of the present study. Moreover, no clear dependency between isoflavones content and the cytotoxic activity of the sprouts was noted [3]. No studies have been published so far concerning lupin sprouts’ impact on breast cancer cells. The only information on the cytotoxic activity of lupin sprouts concerned 5-day sprouts harvested in darkness, with no activity on leukemia HL-60 cells up to 100 µg/mL [52]. The cytotoxic activity of individual isoflavones, which were present in the examined extracts in the highest amount, namely formononetin and biochanin A, was recently reviewed. Notably, formononetin revealed only a weak cytotoxic impact on MCF7 and MDA-MB-231 breast cancer cells, with IC_50_ in the range 50–100 µM or, most often, exceeding the latter value [53], while biochanin A was much more active on the mentioned cell lines, with IC_50_ 5 and 10 µM, respectively [54].

#### 2.4.2. Influence of Chickpea and Lupin Sprouts on Prostate Cells’ Viability

**Figure 5:**
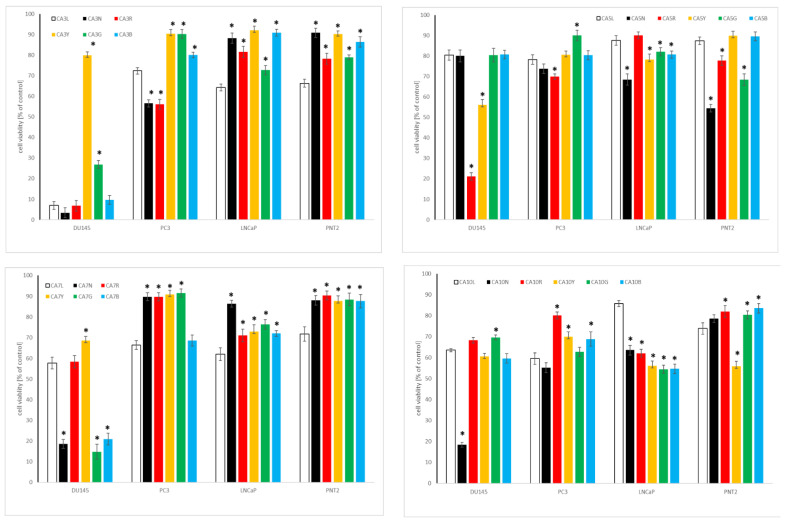
Cytotoxic effect of the extracts of chickpea sprouts (CA) harvested for 3, 5, 7, and 10 days in different LED light conditions: natural (L), darkness (N), red (R), yellow (Y), green (G), blue (B) on prostate normal (PNT2) and cancer (DU145, PC3, LNCaP) cells. Cells were treated with 500 µg/mL of sprout extracts (n = 3) for 24 h. Values are presented as the mean ± SD (standard deviation). Significant differences (*p* < 0.05) for each cell line refer to normal sprouts harvesting procedure and are marked as *.

**Figure 6:**
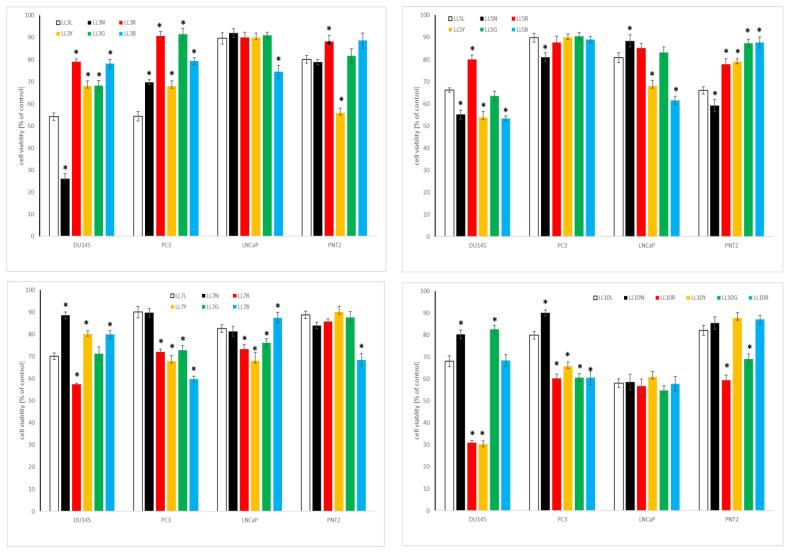
Cytotoxic effect of the extracts of lupin sprouts (LL) harvested for 3, 5, 7, and 10 days in different LED light conditions: natural (L), darkness (N), red (R), yellow (Y), green (G), blue (B) to prostate normal (PNT2) and cancer (DU145, PC3, LNCaP) cells. Cells were treated with 500 µg/mL of sprout extracts (n = 3) for 24 h. Values are presented as the mean ± SD (standard deviation). Significant differences (*p* < 0.05) for each cell line refer to normal sprouts harvesting procedure and are marked as *.

The results of the cytotoxic impact of the tested sprouts on the cells within the prostate panel is presented in Table 3 as IC_50_, and in Figure 5 and Figure 6, for the highest tested concentration of 500 µg/mL. The only activity was noted towards low metastatic and androgen-independent DU145 prostate cancer cells, with the predominance of CA sprouts over LL sprouts extracts. The observed activity can be classified as moderate, for CA10L, CA7N, CA10N, and CA5G, with IC_50_ ranging from 102.1 to 179.8 µg/mL, or as weak, for CA5N, CA10Y, CA10G, and CA5B, with IC_50_ of 205.6–427.5 µg/mL, according to NCI [49]. As far as lupin sprouts are concerned, only weak activity was noted for LL3N, LL10R, and LL10Y, with IC_50_ ≥ 360 µg/mL.

None of the tested extracts were active against androgen-sensitive LNCaP, nor to highly metastatic and androgen-independent PC3 prostate cancer cells. At the highest tested concentration, cell viability was about 60% for CA10 and LL10 sprouts. Importantly, the vast majority of the tested extracts were not toxic to non-neoplastic prostate epithelial cells PNT2, with cell viability above 70%. No studies have been so far published concerning the activity of chickpea or lupin sprouts on prostate cancer cells. As far as the effect of cytotoxic properties of individual isoflavones, predominating in the examined extracts, on prostate cancer cells are concerned, formononetin was moderately or weakly active to DU145, PC3, and LNCaP cells, with IC_50_ 50–100, 25–80, and 40–80 µM, while for biochanin A the value was in a range 50–100 µM [53,54]. The effects observed for individual isoflavones and those for the examined extracts may suggest the enhancement of the activity for the isoflavones in the extract, but also the impact of other phytochemicals (e.g., saponins, phenolic acids) should be taken into account. However, this speculation necessitates further study.

### 2.5. Chemometric Analysis

**Figure 7:**
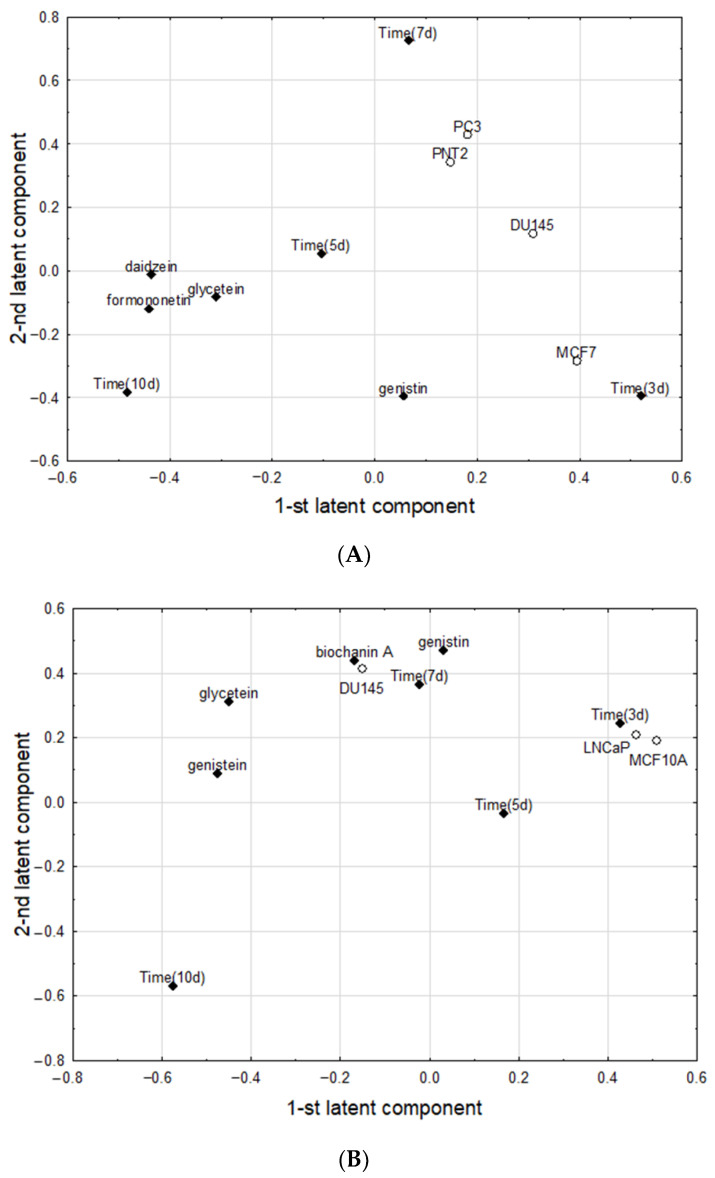
The parameter loadings on first and second latent components in the first PLS model (the predictive parameters are marked with diamonds, and response parameters by circles); (**A**) for chickpea sprouts; (**B**) for lupin sprouts. Time (3d), (5d), (7d), (10d)—sprouts harvested for 3, 5, 7, and 10 days.

Two PLS models fulfilling cross-validation criteria were constructed. The first one (for the experiment with chickpea sprouts) had two significant latent components, with eigenvalues of 2.95 and 1.38, and explained 54.1% of variance in the predictive parameters and 33.5% of variance in the response parameters, respectively. The parameter loadings on the first and second latent components in the first PLS model were shown in Figure 7A. The first latent component in this model had positive loadings predominantly for the Time (3d) and MCF7, and negative loadings for daidzein, formononetin, and Time (10d). The second latent component was loaded mainly positively by Time (7d), PC3, and PNT2. The set of significant correlation weights with absolute values higher than 0.0100, was shown in Table 4.

The second PLS model (for the experiment with lupin) had four significant latent components, with first two having eigenvalues of 2.83 and 2.22, and explaining 63.1% of variance in the predictive parameters and 60.5% of variance of in the response parameters, respectively. The parameter loadings on first and second latent components in the second PLS model were shown on Figure 7B. The first latent component in this model had positive loadings predominantly for the Time (3d), LNCaP, and MCF10, which were mutually highly positively correlated, and, simultaneously, negatively correlated with glycitein and genistein, which, apart from Time (10d) loaded negatively on the same latent component. The second latent component was loaded mainly positively by genistein, biochanin A, Time (7d), DU145 and glycitein, being in one cluster of mutually strongly correlated parameters. Two parameters from this cluster, with the highest positive loadings on the second latent component (genistein, biochanin A) correlated negatively with Time (10d), which deviated from other parameters and alone loaded negatively on the second latent component. The set of significant correlation weights with absolute values higher than 0.100 was shown in Table 4. Other parameters, not included in the above models, were considered noninformative, as they did not load significantly on latent components and therefore did not correlate significantly with other parameters.

## 3. Materials and Methods

### 3.1. Plant Materials and Growth Conditions in LED Chambers

Six species of legume seeds were chosen for harvesting: lupin (*Lupinus luteus* L. var. MISTER); common vetch (*Vicia sativa* L. var. HANKA); bird’s-foot (*Ornithopus sativus* Brot.); lotus (*Lotus corniculatus* L.); chickpea (*Cicer arietinum* L.); and lentil (*Lens culinaris* Medik.). The first four were purchased in Małopolska Hodowla Roślin (Kraków, Poland), and the other two species were purchased in Bavicchi Geo (Perugia, Italy). All evaluated seeds were stored in the Department of Food Chemistry and Nutrition seeds collection, with appropriate voucher numbers: LL/PP/PL1048; CA/PP/PL1051; VS/PP/Pl1046; LENC/PP/PL1050; OS/PP/PL1047 and LC/PP/PL1049, respectively.

The seeds were washed with distilled water and soaked in water for 3 h at room temperature. Then, the seeds were spread in the automatic sprouts EQMM Easy Green Microfarm, and grown for 3, 5, 7, and 10 days after seeding, at 22 ± 2 °C, 70% humidity, in different LED lights: red (R), blue (B), green (G) and yellow (Y), in total darkness (N) exposure (24 h/day), and with natural condition day/night (L) as the control reference sprouts. All sprouts were watered 3 times a day. The obtained samples were denoted using the acronyms of their Latin names: LL for lupin, CA for chickpea, vs. for common vetch, LENC for lentil, OS for bird’s foot, and LC for lotus, with appropriate numbers indicating the cultivation period and letters for quality of light, as described above (e.g., CAL means chickpea sprouts grown in natural condition day/night). The seeds (S) and the collected sprouts were frozen, at −20 °C awaiting further analysis. For each of the treatments, three replicates were taken for analysis.

### 3.2. Reagents

Dimethyl sulfoxide (DMSO), chloroform, HPLC grade acetonitrile, water, and formic acid were purchased from Sigma-Aldrich (Seelze, Germany). Reference standards for HPLC analysis of isoflavones: biochanin A, formononetin, genistein, genistein, glycitein, daidzein, ononin, were purchased from Fluka Chemie (Buchs, Switzerland). Methanol was from Avantor Performance Materials Poland S.A. (Gliwice, Poland). All reagents were of analytical grade. Distilled water was purchased from Sigma-Aldrich (Seelze, Germany). Cell culture media and supplements: DMEM/F12, DMEM low glucose, RPMI1640, MEM, non-essential amino acids (NEAA), sodium pyruvate, epidermal growth factor (EGF), insulin, hydrocortisone, cholera toxin, fetal bovine serum (FBS), donor horse serum, antibiotics solution (10,000 U penicillin and 10 mg streptomycin), phosphate buffered saline (PBS), trypsin solution, were purchased from Sigma-Aldrich (Seelze, Germany).

### 3.3. Extract Preparation

The seeds and sprouts were Soxhlet extracted with methanol for 3 h. After the extraction process, the obtained methanol extracts were decanted, centrifuged, and stored in a freezer at −20 °C prior to HPLC isoflavones analysis. The methanol extracts were further evaporated, and the dry residues were dissolved in DMSO and used for the determination of cytotoxicity.

### 3.4. Isoflavones Analysis

The quantitative analysis of isoflavones was performed, as previously described [3], using the Dionex HPLC system, equipped with a PDA 100 UV-VIS detector and a Hypersil Gold (C-18) column (5 μm, 250 × 4.6 mm, Thermo EC). Analysis was carried out in gradient mode, with 1% aqueous solution of formic acid (A) and acetonitrile (B), 5–60% B in 60 min, at a flow rate of 1 mL/min, with the detection wavelengths 254 and 285 nm. The compounds were identified by comparing their retention times and UV spectra with those of the reference standards (biochanin A, daidzein, daidzein, formononetin, genistein, genistein, glycitein, ononin). The isoflavones content was calculated by measuring the peak area with respect to the standard curves (prepared from the standards at the range 0.0625–1 mg/mL). All analyses were performed in three independent experiments, and the mean value was expressed in mg/100 g of dw. Additionally, the sum of the isoflavones amount was calculated.

### 3.5. Cell Cultures

Human cancer and normal cells: androgen-insensitive prostate carcinoma DU-145, derived from the metastatic site: brain, ATCC HTB-81; androgen-insensitive, grade IV prostate carcinoma, PC-3, derived from the metastatic site: bone, ATCC CRL-1435; androgen-sensitive prostate adenocarcinoma LNCaP, derived from the metastatic site: lymph node, ATCC CRL-1740; prostate epithelial cells, PNT2, ECACC 95012613, ER-positive breast adenocarcinoma MCF7, ATCC HTB-22; ER-negative breast adenocarcinoma MDA-MB-231, ATCC HTB-26; breast epithelial MCF10A, ATCC CRL-10317 were grown under standard conditions (37 °C, 5% CO_2_, relative humidity) and culture media (DMEM/F12 for PNT2, PC3, MDA-MB-231; DMEM Low Glucose for DU145; RPMI1640 with sodium pyruvate for LNCaP; MEM with NEAA for MCF7; DMEM/F12 with 20 ng/mL epidermal growth factor (EGF), 10 µg/mL insulin, 0.5 µg/mL hydrocortisone, 100 ng/mL cholera toxin for MCF10A), supplemented with 10% fetal bovine serum (FBS) or 5% donor horse serum for MCF10A, and 1% antibiotics solution (10,000 U penicillin and 10 mg streptomycin/mL). All cell lines were purchased from Sigma-Aldrich (Seelze, Germany).

### 3.6. Cytotoxic and Viability Assay

Cell viability was determined after 24 h of incubation by MTT assay, as previously described [55]. The examined extracts were dissolved in DMSO, and then diluted in the culture medium to the appropriate concentrations (from 0 to 500 μg/mL). The absorbance was measured at 570 nm using a Biotek Synergy microplate reader (BioTek Instruments Inc., Winooski, VT, USA). Three independent experiments were performed, and the results are expressed as cell viability as % of the control, untreated cells (mean ± SD), and IC_50_ values (concentration at which viability is inhibited by 50 percent).

### 3.7. Statistical Analysis

In this study, the data were expressed as mean ± standard deviation (SD) and were analyzed using a one-way analysis of variance (ANOVA), along with a post-hoc Tukey’s test.

#### Chemometric Method

Partial Least Square (PLS) models were used to reveal the correlation structure between the investigated parameters. The mathematical details of the PLS method are described elsewhere [56,57]. The parameters with large absolute values of their loadings (>0.3) in the PLS model were assumed to be correlated. The parameters were considered negatively correlated if their loadings within the PLS model showed the opposite signs; otherwise, they were considered positively correlated. To express the strength of bivariate associations for the pairs of correlated parameters, the correlation weights were calculated. The details of our approach to PLS models were described in several previous papers [58,59,60]. Statistical analyses were carried out using package SIMCA-P v.9 (Umetrics, Umeå, Sweden). The correlation weights were calculated using software by MP System Co. (Kraków, Poland). The package STATISTICA v. 13.3. (TIBCO Software Inc., Palo Alto, CA, USA) was used for graphic representation of data.

## 4. Conclusions

In summary, our results indicate that yellow LED light exposure was optimal for inducing the accumulation of isoflavones in 10-day chickpea sprouts, with ononin as the predominant compound, while for green LED light the isoflavones sum was the lowest. The exact opposite observations were made for lupin sprouts, with the predominance of green over the yellow LED light. This may imply that the observed effect of LED light of different quality is genus- or even species-specific; however, further studies are needed on more sprout species. Moreover, with isoflavones’ sum up to almost 1 g/100 g dw (translating approximately to 250 mg/100 g in fresh sprouts), chickpea sprouts are a rich, novel source of dietary isoflavones, comparable with, or even better than commercially available and popular soy (up to 500 mg of isoflavones/100 g dw) or alfalfa (up to 200 mg of isoflavones/100 g dw) sprouts [3].

The examined sprouts were of high safety to non-neoplastic breast and prostate cells, with interesting cytotoxic effects on some of the tested cancer cells. However, further research is required to adequately explain the effects and relationship between the isoflavones content and cytotoxic activity of the examined sprouts against hormone-dependent cancer cells, especially those with overexpression of estrogen receptors. Some studies on other cellular functions, including proliferation, are especially needed. The developed model of evaluation provides a new opportunity to manipulate the content of bioactive compounds in the sprouts, intended for consumers with special chemopreventive needs, including menopause disorders or the risk of hormone-dependent cancers.

## Figures and Tables

**Table 1:** Content (mg/100 g dw) of individual isoflavones and their sums in chickpea sprouts (CA) harvested in different LED light conditions for 3, 5, 7, and 10 days (mean ± SD; n = 3).

Time of Sprouting	Type of Light	Biochanin A	Daidzein	Formononetin	Genistein	Glycitein	Ononin	Sum of Isoflavones
CA3	Natural	0.39 ± 0.16	0.64 ± 0.13	Tr	8.51 ± 0.21	4.90 ± 0.50	Tr	14.44
CA5		49.0 ± 5.14	1.91 ± 0.16	79.7 ± 6.2	1.05 ± 0.18	2.42 ± 0.12	443.3 ± 18.3	577.4
CA7		154.5 ± 11.4	2.14 ± 0.21	267.6 ± 21.9	0.91 ± 0.10	5.73 ± 0.70	51.9 ± 2.8	482.8
CA10		309.8 ± 29.8	3.38 ± 0.21	369.6 ± 23.5	3.02 ± 0.26	8.32 ± 0.36	97.2 ± 13.6	791.3
CA3	Without light	67.10 ± 4.2	0.53 ± 0.15	96.8 ± 8.7	Tr	1.64 ± 0.17	25.4 ± 0.7	191.5
CA5		100.3 ± 5.0	1.11 ± 0.06	177.9 ± 7.0	0.36 ± 0.09	2.99 ± 0.10	31.9 ± 0.7	314.6
CA7		152.6 ± 4.6	2.02 ± 0.13	211.2 ± 15.7	0.86 ± 0.11	5.00 ± 0.35	66.4 ± 3.7	438.1
CA10		144.3 ± 7.9	1.57 ± 0.09	165.7 ± 14.9	1.00 ± 0.15	4.52 ± 0.16	242.5 ± 8.5	559.6
CA3	Red	61.6 ± 13.4	0.34 ± 0.07	82.4 ± 10.4	0.16 ± 0.04	0.93 ± 0.20	30.2 ± 4.9	175.6
CA5		67.16 ± 4.1	0.58 ± 0.10	109.2 ± 9.5	0.81 ± 0.05	1.51 ± 0.11	454.0 ± 35.8	633.3
CA7		166.9 ± 13.0	1.79 ± 0.12	182.6 ± 11.4	1.76 ± 0.04	4.46 ± 0.22	413.5 ± 16.7	771.0
CA10		44.2 ± 11.9	0.99 ± 0.06	31.9 ± 2.9	Tr	2.65 ± 0.17	6.88 ± 0.60	86.6
CA3	Yellow	21.98 ± 1.84	Tr	36.2 ± 2.8	0.19 ± 0.12	0.28 ± 0.01	169.0 ± 5.5	227.7
CA5		110.4 ± 8.4	0.56 ± 0.12	109.6 ± 18.2	0.56 ± 0.04	1.74 ± 0.10	305.2 ± 7.0	528.1
CA7		124.8 ± 18.1	1.04 ± 0.14	136.4 ± 14.7	1.15 ± 0.11	2.61 ± 0.11	458.5 ± 27.0	724.5
CA10		293.9 ± 26.1	1.57 ± 0.10	227.0 ± 12.8	1.67 ± 0.15	4.11 ± 0.31	425.7 ± 6.6	953.9
CA3	Green	19.6 ± 1.6	Tr	108.0 ± 7.9	Tr	1.95 ± 0.12	24.6 ± 0.9	154.2
CA5		78.5 ± 7.2	0.77 ± 0.09	22.1 ± 2.1	Tr	0.43 ± 0.02	73.1 ± 9.3	174.9
CA7		171.1 ± 6.9	0.62 ± 0.11	54.7 ± 6.5	0.26 ± 0.02	4.50 ± 0.23	28.4 ± 7.9	259.6
CA10		54.7 ± 5.5	0.32 ± 0.08	46.7 ± 1.4	0.15 ± 0.01	0.87 ± 0.07	11.8 ± 0.8	114.5
CA3	Blue	25.2 ± 3.5	0.33 ± 0.09	40.8 ± 3.2	Tr	0.67 ± 0.02	14.6 ± 1.1	81.6
CA5		98.9 ± 6.6	0.76 ± 0.13	133.5 ± 12.4	Tr	1.89 ± 0.10	28.6 ± 1.3	263.7
CA7		182.6 ± 13.8	1.26 ± 0.14	272.3 ± 15.1	Tr	2.76 ± 0.23	54.1 ± 3.3	513.0
CA10		257.2 ± 14.8	0.71 ± 0.16	275.1 ± 13.2	Tr	6.52 ± 0.65	23.9 ± 0.9	563.4

Tr—traces.

**Table 2:** Content (mg/100 g dw) of individual isoflavones and their sums in lupin sprouts (LL) harvested in different LED light conditions for 3, 5, 7, and 10 days (mean ± SD; n = 3).

Time of Sprouting	Type of Light	Biochanin A	Genistein	Genistin	Glycitein	Sum of Isoflavones
LL3	Natural	0.21 ± 0.02	Tr	21.80 ± 0.44	3.03 ± 010	25.0
LL5		0.59 ± 0.11	Tr	30.82 ± 0.74	10.95 ± 1.28	42.4
LL7		1.09 ± 0.13	Tr	66.85 ± 2.30	9.20 ± 0.69	77.1
LL10		1.91 ± 0.16	Tr	67.42 ± 2.06	18.04 ± 0.78	87.4
LL3	Without light	0.14 ± 0.02	Tr	17.60 ± 0.43	1.81 ± 0.07	19.5
LL5		1.24 ± 0.13	Tr	11.93 ± 0.64	7.23 ± 0.15	20.4
LL7		8.40 ± 0.48	17.67 ± 2.21	147.2 ± 3.6	31.31 ± 1.04	204.6
LL10		1.13 ± 0.11	42.43 ± 1.48	18.60 ± 0.54	27.37 ± 1.64	89.5
LL3	Red	0.79 ± 0.14	18.00 ± 0.94	42.60 ± 1.42	13.75 ± 0.17	75.1
LL5		2.43 ± 0.31	6.42 ± 0.65	107.1 ± 6.1	12.65 ± 0.36	128.6
LL7		4.00 ± 0.24	10.41 ± 0.30	119.9 ± 2.6	16.20 ± 0.56	150.5
LL10		1.00 ± 0.33	19.35 ± 3.00	34.27 ± 1.00	17.97 ± 0.74	72.6
LL3	Yellow	2.41 ± 0.20	6.72 ± 1.95	56.90 ± 3.92	9.78 ± 0.45	75.8
LL5		3.63 ± 0.20	6.60 ± 0.20	89.19 ± 1.75	7.54 ± 0.22	107.0
LL7		2.69 ± 0.20	13.38 ± 1.04	79.14 ± 1.32	11.90 ± 0.43	107.1
LL10		1.38 ± 0.18	12.95 ± 2.60	15.60 ± 0.53	12.72 ± 0.41	42.6
LL3	Green	0.45 ± 0.08	5.54 ± 0.74	29.31 ± 0.74	10.42 ± 0.20	45.7
LL5		0.44 ± 0.20	6.05 ± 0.12	37.54 ± 1.90	10.62 ± 0.35	54.6
LL7		2.83 ± 0.20	13.37 ± 0.23	63.36 ± 2.15	10.75 ± 0.60	90.3
LL10		3.10 ± 0.40	46.22 ± 1.65	33.86 ± 1.21	42.69 ± 1.21	125.9
LL3	Blue	4.11 ± 0.19	4.29 ± 0.08	21.80 ± 1.00	12.29 ± 1.05	42.5
LL5		0.87 ± 0.17	9.10 ± 0.78	22.81 ± 1.12	3.42 ± 0.28	36.2
LL7		3.14 ± 0.26	12.36 ± 0.78	50.05 ± 4.40	14.27 ± 2.56	79.8
LL10		4.09 ± 0.28	31.66 ± 0.33	19.50 ± 2.60	24.11 ± 1.18	79.4

Tr—traces.

**Table 3:** Cytotoxic activity of the chickpea (CA) and lupin (LL) seeds and sprouts harvested for 3, 5, 7, and 10 days on prostate cancer (DU145) and breast cancer (MDA-MB-231, MCF7) cells, expressed as IC_50_ values (µg/mL).

Samples	DU145	MCF7	Samples	DU145	MDA-MB-231	MCF7
CA3L	>Cmax	>Cmax	LL3L	>Cmax	>Cmax	>Cmax
CA5L	>Cmax	418.6	LL5L	>Cmax	>Cmax	198.2
CA7L	>Cmax	214.5	LL7L	>Cmax	>Cmax	111.6
CA10L	102.1	35.0	LL10L	>Cmax	>Cmax	77.2
CA3N	>Cmax	148.6	LL3N	360.0	>Cmax	202.9
CA5N	205.6	42.1	LL5N	>Cmax	>Cmax	152.8
CA7N	173.5	133.4	LL7N	>Cmax	>Cmax	174.6
CA10N	117.4	51.6	LL10N	>Cmax	>Cmax	>Cmax
CA3R	302.1	484.9	LL3R	>Cmax	>Cmax	>Cmax
CA5R	>Cmax	183.6	LL5R	>Cmax	>Cmax	>Cmax
CA7R	>Cmax	178.6	LL7R	>Cmax	>Cmax	201.9
CA10R	>Cmax	166.2	LL10R	375.5	>Cmax	370.5
CA3Y	>Cmax	317.9	LL3Y	>Cmax	>Cmax	187.1
CA5Y	>Cmax	>Cmax	LL5Y	>Cmax	>Cmax	154.8
CA7Y	>Cmax	93.8	LL7Y	>Cmax	>Cmax	399.6
CA10Y	343.4	70.6	LL10Y	360.4	>Cmax	>Cmax
CA3G	>Cmax	>Cmax	LL3G	>Cmax	>Cmax	>Cmax
CA5G	179.8	193.0	LL5G	>Cmax	>Cmax	419.9
CA7G	>Cmax	171.4	LL7G	>Cmax	320.6	292.0
CA10G	288.3	137.8	LL10G	>Cmax	>Cmax	142.8
CA3B	>Cmax	301.8	LL3B	>Cmax	>Cmax	424.1
CA5B	352.0	332.2	LL5B	>Cmax	>Cmax	164.2
CA7B	>Cmax	67.2	LL7B	>Cmax	307.5	282.2
CA10B	427.5	46.8	LL10B	>Cmax	>Cmax	145.9
CAS	154.1	120.5	LLS	>Cmax	>Cmax	>Cmax

**Table 4:** Correlation weights for the pairs of parameters based on the PLS model.

Pairs of Correlated Parameters		Correlation Weights
	Chickpea sprouts	
Time (7d)	PC3	0.295
Time (7d)	PNT2	0.235
Time (3d)	MCF7	0.205
Time (10d)	Formononetin	0.195
Time (10d)	Daidzein	0.168
PNT2	PC3	0.146
Time (10d)	Glycitein	0.137
Time (3d)	Genistein	0.110
Formononetin	Du145	−0.109
Glycitein	MCF7	−0.114
Daidzein	Du145	−0.124
Daidzein	MCF7	−0.142
Genistein	PC3	−0.144
Time (3d)	Glycitein	−0.148
Formononetin	MCF7	−0.168
Time (3d)	Daidzein	−0.186
Time (10d)	MCF7	−0.190
Time (3d)	Formononetin	−0.210
Time (7d)	Genistein	−0.277
	Lupin sprouts	
LNCaP	MCF10A	0.237
Time (3d)	MCF10A	0.215
Time (3d)	LNCaP	0.197
Genistein	DU145	0.185
Biochanin A	DU145	0.180
Time (7d)	Genistein	0.170
Time (10d)	Genistein	0.159
Time (7d)	Biochanin A	0.152
Time (7d)	DU145	0.143
Glycitein	DU145	0.107
Time (10d)	Biochanin A	−0.100
Time (10d)	MCF10A	−0.124
Time (10d)	Genistein	−0.174
Time (3d)	Glycitein	−0.191
Time (3d)	Genistein	−0.192
Glycitein	LNCaP	−0.205
Genistein	LNCaP	−0.214
Glycitein	MCF10A	−0.222
Genistein	MCF10A	−0.239

Time (3d), (7d), (10d)—sprouts harvested for 3, 5, 7, and 10 days.

## Data Availability

On request.

## Supplementary Materials

The following supporting information can be downloaded at: https://www.mdpi.com/article/10.3390/molecules27249030/s1, Supplementary Figure S1: Diagrams of evaluated isoflavones transformation in chickpea sprouts in relation to the different light treatments. Supplementary Figure S2: Diagrams of evaluated isoflavones transformation in lupin sprouts in relation to the different light treatments.

## Sample Availability

Samples of the extracts are available from the authors.
