# Supplementary material for: Chickpea and Lupin Sprouts, Stimulated by Different LED Lights, As Novel Examples of Isoflavones-Rich Functional Food, and Their Impact on Breast and Prostate Cells
Source: Molecules. 2022 Dec 18;27(24):9030. doi: 10.3390/molecules27249030 (PMC9781113; doi:10.3390/molecules27249030)

Supplementary Figure S1. Diagrams of evaluated isoflavones transformation in chickpea sprouts in relation to the different light treatments.

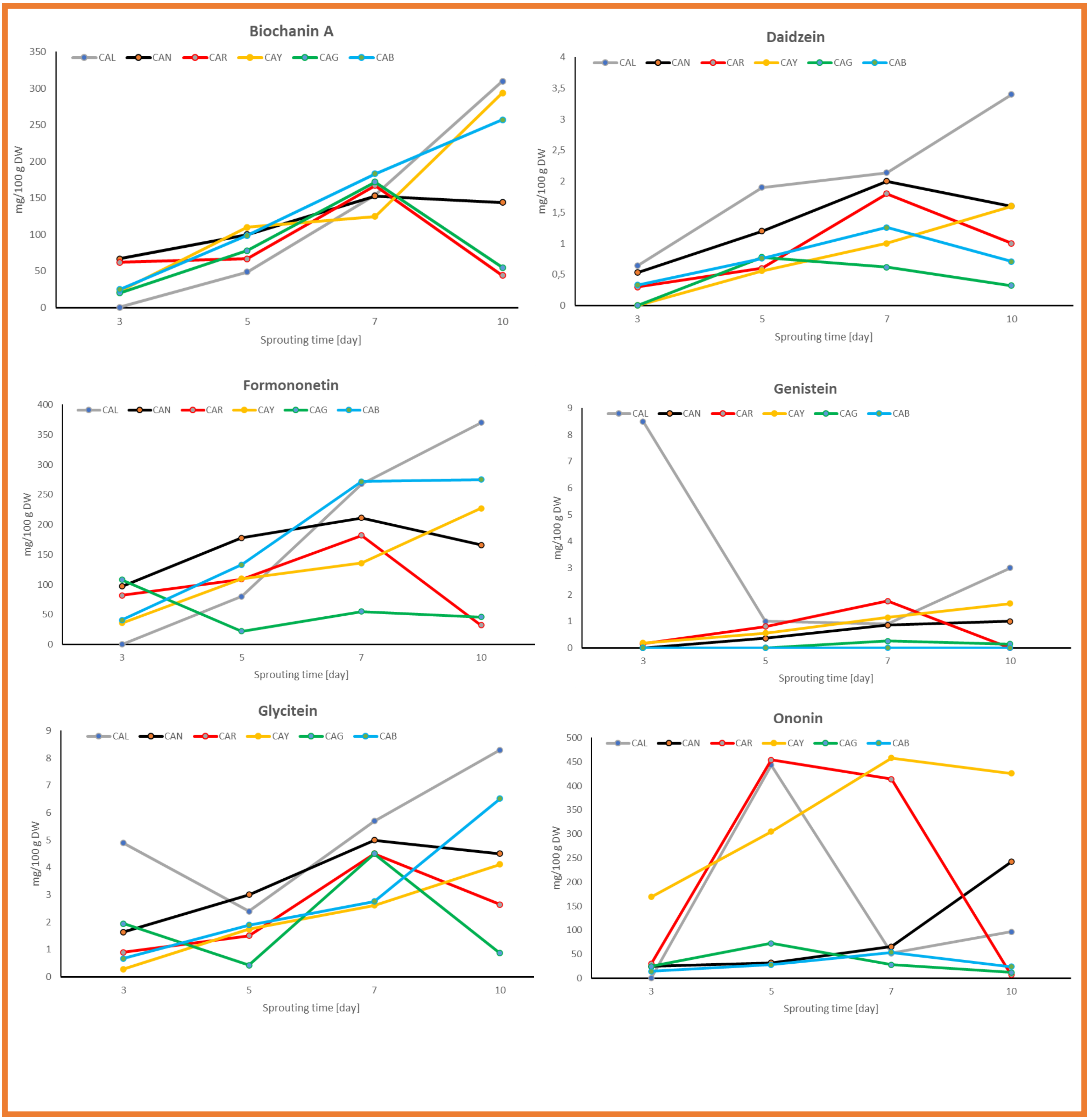

Supplementary Figure S2. Diagrams of evaluated isoflavones transformation in lupin sprouts in relation to the different light treatments.

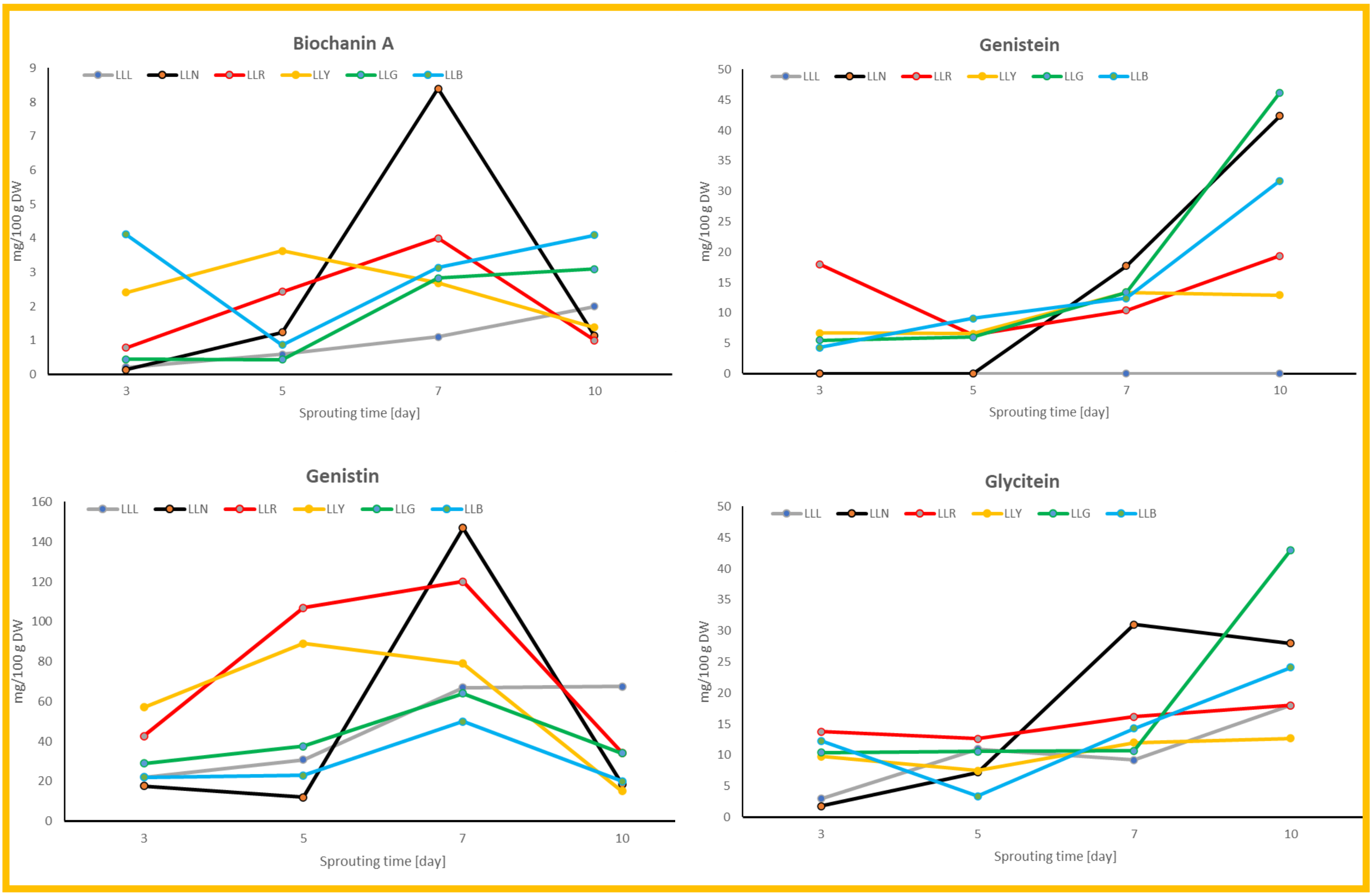

Supplement: Supplementary file 1 [file molecules-27-09030-s001.zip › molecules-2086577-SI.pdf]
